# Physico-chemical and Textural Properties of 3D Printed Plant-based and Hybrid Soft Meat Analogs

**DOI:** 10.1007/s11130-023-01068-4

**Published:** 2023-05-18

**Authors:** Tianxiao Wang, Lovedeep Kaur, Akashdeep Singh Beniwal, Yasufumi Furuhata, Hiroaki Aoyama, Jaspreet Singh

**Affiliations:** 1 School of Food and Advanced Technology, Massey University, Palmerston North, New Zealand; 2 Riddet Institute, Massey University, Palmerston North, New Zealand; 3 Ajinomoto Co., Inc, Suzuki-cho 3-1, Kawasaki-ku, Kawasaki-shi, Japan

**Keywords:** Food 3D printing, Hybrid meat analogs, Texture, Microstructure, Pea protein, Chicken

## Abstract

This study investigated the physico-chemical and textural properties of 3D-printed pea protein-only and pea protein-chicken-based hybrid meat analogs. Both pea protein isolate (PPI)-only and hybrid cooked meat analogs had a similar moisture content of approximately 70%, which was similar to that of chicken mince. However, the protein content increased significantly with the amount of chicken in the hybrid paste undergoing 3D printing and cooking. Significant differences were observed in the hardness values of the non-printed cooked pastes and the 3D printed cooked counterparts, suggesting that the 3D printing process reduces the hardness of the samples and is a suitable method to produce a soft meal, and has significant potential in elderly health care. Scanning electron microscopy (SEM) revealed that adding chicken to the plant protein matrix led to better fiber formation. PPI itself was not able to form any fibers merely by 3D printing and cooking in boiling water. Protein-protein interactions were also studied through the protein solubility test, which indicated that hydrogen bonding was the major bonding that contributed to the structure formation in cooked printed meat analogs. In addition, disulfide bonding was correlated with improved fibrous structures, as observed through SEM.

**Supplementary Information:**

The online version contains supplementary material available at 10.1007/s11130-023-01068-4.

## Introduction

Three-dimensional (3D) printing is a novel manufacturing technology that is gaining a lot of attention for its application in the food industry. It has been used in the construction, medical, and aerospace industry utilizing diverse materials such as plastic, metal biopolymers etc. [1–4]. 3D printing creates unique geometric shapes using three-dimensional computer-aided design (CAD). Therefore, the 3D structures are built through the layer-by-layer deposition of material inks which is fed into the 3D printers and squeezed from the nozzles of printers [4]. There are various categories of 3D printing techniques explained in detail by Tejo-Otero and team [4].

Meat analogs are generally made from vegetables or plants rich in protein and are considered a replacer by mimicking the characteristics of meat. Currently, techniques like high moisture extrusion and shear cell are used to create fibrous structures in plant protein dispersions through shear and mixing. The use of 3D printing for manufacturing meat analogs has been explored in recent years [5–7], showing the feasibility of printing products that look like meat by using plant-based materials. Currently, there is limited available literature on the characterization of 3D-printed meat analogs [8]. However, the physico-chemical properties of meat analogs after cooking were not evaluated in that study. According to previous research, textural profiles, nutrient contents, microstructure, and protein-protein interactions are major properties of meat analogs, which have been frequently measured through instruments [9–12].

Previous studies have indicated that the textural properties of 3D printed products are related to the food composition and nozzle size [1, 13]. Huang et al. [13] pointed out that the 3D slicer design was changed by the nozzle size setting. A smaller nozzle diameter resulted in more layers in the sliced objective, which decreased the hardness of printed products. To produce texturally modified foods, it is significant to know how food formulation or printing performance influences food texture. Liu et al. [2] highlighted the relationship between ingredients, printing ability, and food microstructure. Their study identified that whey protein isolate helps create smooth interfaces and increased the stability of printed milk powder. Pea protein has gained a lot of popularity recently as an alternative protein source [14]. A companion paper by the authors [15] describes the detailed process of printing nugget-shaped soft meat analogs by using pea protein isolate (PPI)-only and PPI-chicken-based formulations. The addition of raw chicken paste to cooked PPI–based paste was recommended as it provides the rheological properties required for 3D printing. The main objective of the current study was to assess and compare the physico-chemical and textural properties of the printed plant-only and hybrid meat analogs.

## Materials and Methods

### Materials

Commercial chicken mince (5% fat and 95% protein) and beef fat (Premium 100% pure beef dripping, Farmland foods, NZ) were purchased from a local supermarket, while PPI (80% protein) was sourced from Davis Ingredient Ltd. (Palmerston North, NZ). Maize starch (pregelatinized, Hi-Maize 1043, National starch and Chemical NZ Ltd.) and soy lecithin (Hawkins Watts Ltd., NZ) were also used.

### Sample Preparation and 3D Printing

Freshly prepared PPI and PPI pastes containing 20 and 50% chicken (replacing PPI, w/w) were used and are referred to as PSF, 20CHK and 50CHK, respectively. Both these pastes also contained the same amounts of added beef fat, starch and soy lecithin. The amount of water added to make the pastes was adjusted considering the moisture content of the chicken mince in order to keep the total moisture content of the pastes the same- approx. 69%. The details of the methodology to prepare the pastes are presented in detail in a companion paper [15].

An assembled LVE 3D printer (NZ-3D Ltd., New Zealand) was used (Supplementary Figure S1). More details of the printing process are provided elsewhere [15]. A 3D chicken nugget model, built using 3D builder (Version 18.0.1931.0; Microsoft Co.), was loaded and sliced by Repetier Host (Version 2.1.4; Hot-World GmbH & Co. KG.). A small nugget shape sample was printed using the pastes at room temperature, with a printing speed of 15 mm/s and 100% infill density. The diameter of the nozzle was 1.54 mm, and the layer height was 1.5 mm. Printed samples were stored in a refrigerator overnight before transferring and sealing in polypropylene bags for cooking in a boiling water bath for 10 min.

### Moisture and Protein Analysis of Printed Products

The moisture contents of the printed and cooked samples were determined by the hot air oven method [16], with minor modifications. Pre-weighed samples were dried in a hot air oven (108 °C) overnight before weighing them again to calculate their moisture content. The Kjeldahl method was used to determine the protein content [17]. A nitrogen-to-protein conversion factor of 6.25 was used for both pea and chicken protein in this study.

### Textural Profile Analysis (TPA) of Printed Chicken and Printed Analogs

**Fig. 1:**
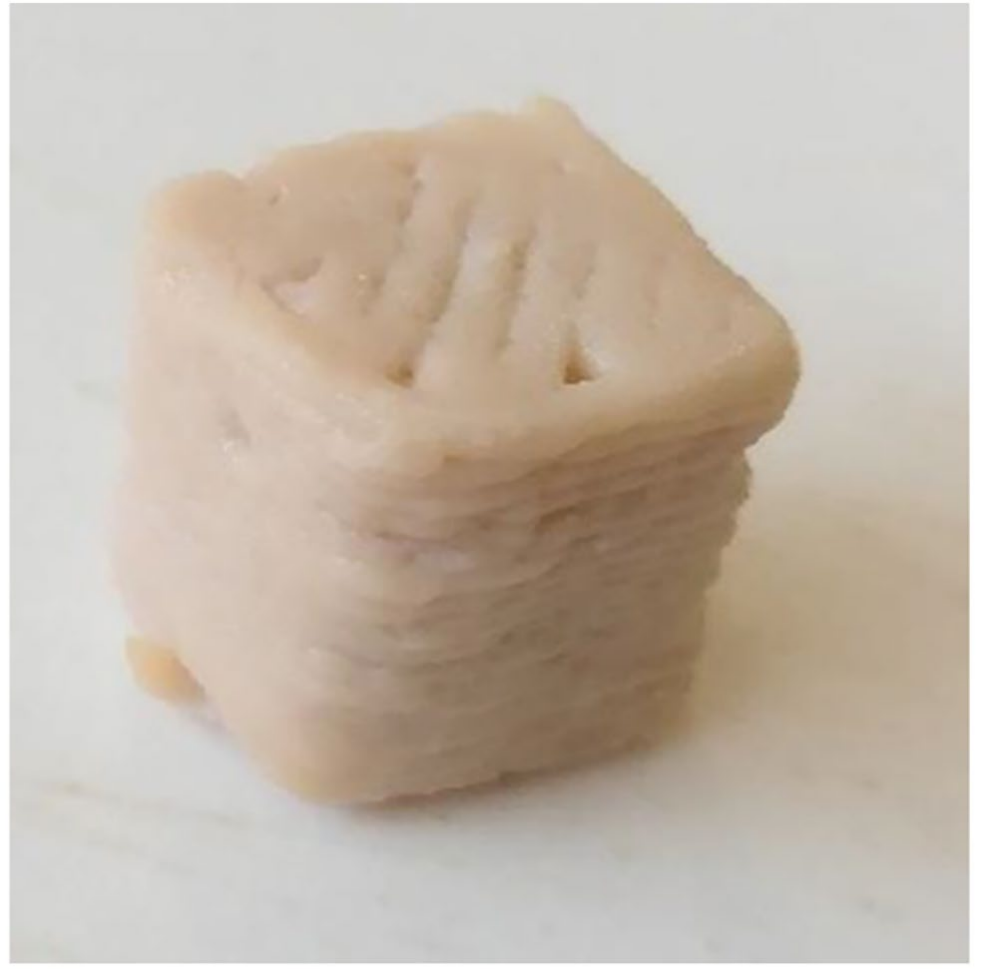
3D printed 20CHK cube

The texture analysis method of printed samples was inspired by Yang et al. [18], who printed samples with a simple cube shape, which was convenient for texture profile analysis. In this study, non-printed samples (including PSF, 20CHK, 50CHK, and chicken mince) were prepared in the same way as described previously. After that, 20CHK, 50CHK, and chicken mince samples were cooked in boiling water for 10 min. Finally, all of them were cut into 2 × 2 × 2 cm^3^ cubes for further analysis. Small cubes of printed PSF, 20CHK, and 50CHK samples were prepared and cooked in the same condition as non-printed samples. Considering the printing defects and cooking shrinkage, a bigger cube size (2.5 × 2.5 × 2.5 cm^3^) was printed (Fig. 1). Then the bigger cubes were cut into 2 × 2 × 2 cm^3^ after cooking. Both printed and non-printed samples were stored at 4 ˚C overnight and kept at room temperature for 30 min before texture analysis.

All samples were positioned on the stage of the Texture Analyser (TA.XT.plus, Stable Micro Systems, UK). The setup and calculation followed the method described by Samard and Ryu [19], with slight modifications. A 61 mm plate cylindrical probe and a load cell with a 50 kg capacity were chosen, and the measurement was based on a double compression test. Each sample was compressed twice with a 50% strain. The speed of the probe was 2 mm/s with 1 mm/s pre-test speed and 5 mm/s post-test speed. The gap time between the two compressions was 5 s. Hardness, springiness, cohesiveness, and chewiness were calculated by the software Exponent (version 6.1.16.0, Stable Micro Systems, UK.

### Microstructure of Chicken and Printed Meat Analogs

Both printed samples and chicken mince were placed in polypropylene bags and cooked for 10 min in boiling water. Non-printed samples were vacuum-packed, while printed samples were packed without vacuum sealing. Cooked chicken and printed meat analogs were fractured by hand. Fractured samples were pre-frozen at -30 ˚C overnight and then freeze-dried in a Cuddon FD18CT freeze drier (Cuddon Blenheim) at -40 ˚C for about 3 days. One tiny piece was taken from the fractured surface of each sample and coated with 100 nm of gold by a sputter coater (Bal-tec SCD 050) for 200 s. The microstructure of coated samples was observed and photographed by an FEI Quanta 200 Environmental scanning electrical microscope (Philips Electron Optics, USA) at 400× magnifications, with an accelerating voltage of 25 kV.

### Protein Interactions (Protein Solubility Test)

Five different extraction solutions were used to dissolve different chemical bonds in the printed samples and pastes (Table 1), as described by Chiang et al. [12], with minor modifications.

**Table 1:** Five extraction solutions prepared by combinations of chemicals and reagents

^1, 2^Extraction solution	Description
PB	0.1 M phosphate buffer (pH 7.5)
PS	PB + 1.5 g/100 mL SDS
PD	PB + 0.05 M DDT
PU	PB + 8 M urea
PSDU	PB + 1.5 g/100 mL SDS + 0.05 M DDT + 8 M urea

^1^ PB represents phosphate buffer; PS represents PB + sodium dodecyl sulfate (SDS); PD represents PB + dithiothreitol (DTT); PU represents PB + urea; PSDU represents PB + SDS + DTT + urea. ^2^ PB with a neutral pH was used as the control extraction solution to dissolve native protein. SDS, DTT, and urea were used to destroy hydrophobic interactions, disulfide bonding, and hydrogen bonding, respectively.

Printed PSF, 20CHK, 50CHK, and non-printed raw PSF paste (0.5 g) were weighed and dissolved with 10 mL of different extraction solutions in centrifugation tubes and shaken for 30 min in a refrigerated incubator shaker. Then they were further blended with the help of a high-speed disperser (Ultra-Turrax® T25 Basic, IKA, Germany) at 4,390 × g for 30 s. and shaken again for another 30 min. Finally, samples were centrifugated in Heraeus™ Multifuge™ X3R centrifuge (Thermo Fisher Scientific, New Zealand) at 3,494 × g for 10 min. The supernatant (5 µL) of each centrifuged sample was pipetted into a microplate with 250 µL Bradford reagent (Thermo Fisher Scientific, New Zealand and the protein amount in supernatants was measured by a microplate reader (SPECTROstar Nano, BMG Labtech, Australia) at 595 nm. The standard absorbance curve was obtained by measuring protein content in bovine serum albumin (BSA). Finally, the percentage of soluble protein was calculated:

$${\rm{\% }}\,{\rm{Solubility}}\,{\rm{ = }}\,\frac{{{\rm{\% }}\,{\rm{soluble}}\,{\rm{protein}}}}{{{\rm{\% }}\,{\rm{total}}\,{\rm{Protein}}}}{\rm{ \times 100}}$$

Where % total protein was measured by the Kjeldahl method [17].

### Statistical Analysis

The data presented in the results and discussions are the mean values of triplicated measurements. One-way analysis of variation (ANOVA) and Tukey’s pairwise comparisons were conducted by Minitab (version 18.1, Minitab Inc., State College, PA) to analyze the significance of the data. Statistical significance was defined by a *p-value* lower than 0.05.

## Results and Discussion

### Moisture and Protein Contents

Moisture content in PSF is lower than in samples with chicken, but there is no significant difference (*p <* 0.05, Table 2). Yao et al. [10] found that extruded meat analogs containing approximately 60% moisture showed a desirable fibrous structure that was not observed in samples with around 70% moisture. It may partly explain why the PSF sample had little fiber formation after printing and cooking. For 3D printing, however, reducing the moisture content of PPI paste would decrease the flowability. PPI paste becomes too dry if the moisture content is reduced to 60%. On the other hand, adding other solid ingredients might cause the paste to be too sticky (According to preliminary experiments [15]).

For meat analogs, protein content is relevant to their nutritional value. In this study, protein content was higher in the sample containing a higher amount of chicken (Table 2). Protein in the boiled chicken sample was nearly 27%, which is significantly higher than in other samples (*p <* 0.05).

**Table 2:** Moisture and protein contents of the cooked 3D-printed samples

^1^Samples	^2,3^Moisture (%)	^2,3^Protein (%)
PSF	69.13 ± 0.40^a^	19.19 ± 0.30^a^
20CHK	70.45 ± 0.43^a^	20.73 ± 0.23^b^
50CHK	70.04 ± 0.43^a^	22.88 ± 0.29^c^
Chicken	70.20 ± 0.90^a^	26.95 ± 0.48^d^

^1^PSF represents PPI paste containing both starch and fat; 20CHK represents 20% chicken added into PSF paste; 50CHK represents 50% chicken added into PSF paste. ^2^Results are shown as means ± SD (n = 3). ^3^different letters in each column show a significant difference (*p <* 0.05).

#### Texture Profile Analysis

The instrumental hardness of food products refers to the force to break food samples by molar teeth [20]. As can be seen in Table 3, all meat analog samples have a significantly lower hardness than cooked chicken mince (*p <* 0.05). The 3D printing process approximately halves the hardness of non-printed meat analogs. The hardness reduction is related to the space between deposition lines in the printing process. The restructured food material contributes a less intensive matrix compared with non-printed samples.

The hardness was reduced with the increasing amount of pea protein paste. Due to all samples containing roughly the same level of moisture, the different values of hardness should be caused by the varying formulations. As stated by previous researchers, pea protein tends to form a soft gel [21, 22]. This could be the reason why the hardness of cooked PSF is approximately 10 times lower than cooked chicken. In addition, the hardness of 50CHK is lower than the average hardness of cooked chicken mince and PSF. The presence of protein-polysaccharides may result in the incompatibility of phases during thermal processing leading to repulsive forces causing a break in restructured PSF blend leading to reduced hardness [23].

Other textural properties, including springiness, cohesiveness, and chewiness, are listed in Table 3. These characteristics of meat analogs are also important as they are often referenced in previous research. Cooked chicken mince showed higher values than printed meat analogs. Meanwhile, these textural values of non-printed meat analogs decreased by lowering the amount of chicken. This indicates that adding chicken positively influences springiness, cohesiveness, and chewiness.

The 50CHK sample showed an increased springiness and a decreased cohesiveness after printing. It is associated with the high void rate in printed 50CHK caused by non-smooth printing behavior. In contrast, the printing process reduced springiness and improved the cohesiveness of 20CHK, which might be because of the lower void rate. As to the PSF sample, both springiness and cohesiveness were significantly changed after printing (*p <* 0.05). It might be because a pastier material such as cooked PSF paste was restructured after being extruded and deposited to form a 3D shape. While cooked pastes with chicken were less influenced post-printing due to their more solid-like nature. The chewiness values are generally related to the hardness of samples since a hard sample also exhibits a high chewiness. However, the chewiness of printed PSF is higher than non-printed PSF. In addition, printed PSF shows a huge variation in chewiness, suggesting that printed PSF should be analyzed under a lower load force.

**Table 3:** Textual profiles of printed and non-printed samples after cooking

^1^Samples		^2,3^Hardness (N)	^2,3^Springiness (%)	^2,3^Cohesiveness (%)	^2,3^Chewiness (N)
Chicken mince		46.99 ± 4.00^e^	87.31 ± 2.53^d^	48.21 ± 1.57^d^	19.82 ± 2.36^d^
50CHK	control	16.14 ± 1.14^d^	72.23 ± 8.82^ cd^	33.55 ± 2.61^c^	3.93 ± 0.75^c^
	printed	8.67 ± 0.99^c^	85.17 ± 10.89^d^	30.85 ± 3.02^bc^	2.26 ± 0.34^bc^
20CHK	control	7.21 ± 0.45^bc^	55.10 ± 2.90^bc^	24.58 ± 1.43^ab^	0.98 ± 0.11^ab^
	printed	3.46 ± 0.62^a^	38.79 ± 6.98^ab^	20.98 ± 3.12^a^	0.27 ± 0.05^a^
PSF	control	4.08 ± 0.28^ab^	29.95 ± 5.32^a^	20.60 ± 0.95^a^	0.25 ± 0.06^a^
	printed	1.23 ± 0.23^a^	68.80 ± 27.2^ cd^	31.86 ± 9.86^bc^	0.29 ± 0.21^a^

^1^ PSF represents PPI paste containing starch and fat; 20CHK represents 20% chicken added into PSF paste; 50CHK represents 50% chicken added into PSF paste. All samples were cooked in boiling water for 10 min. ^2^Results were means ± SD (n = 5), obtained from Exponent. ^3^different letters in each column show a significant difference (*p <* 0.05).

### Microstructure of Cooked Chicken and Printed Meat Analogs

**Fig. 2:**
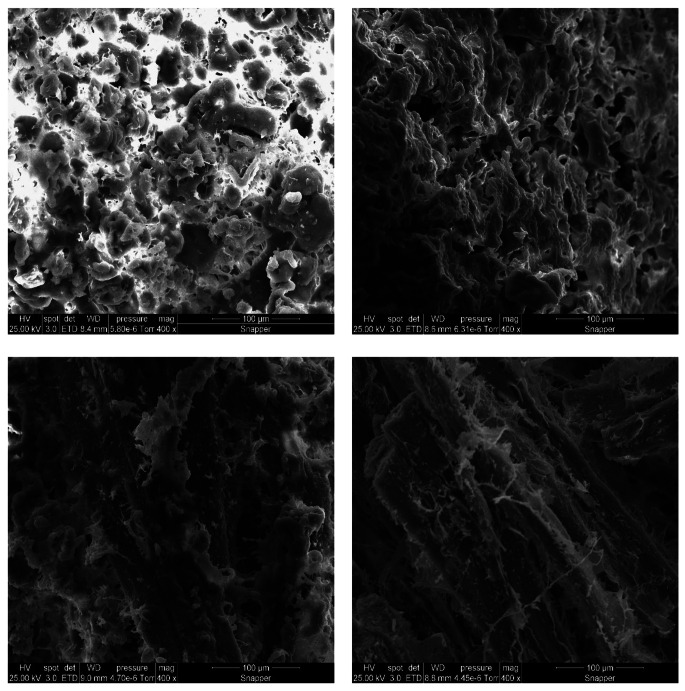
Microstructure of cooked printed meat analogs (PSF (top left), 20CHK (top right), 50CHK (lower left)) and non-printed chicken mince (lower right) viewed by SEM at 400×magnification PSF represents PPI paste containing starch and fat; 20CHK and 50CHK represent 20 and 50% chicken added into PSF paste.

The photos of printed uncooked and cooked meat analogs are shown elsewhere [15]. The PSF was inadequate to form a fibrous structure. More fibers were found in the cooked 20CHK than 50CHK sample. It might be because of a smoother printing flow of 20CHK. Printed PSF showed a highly aggregated microstructure with globular particles inside (Fig. 2). The aggregation is assumed to consist of PPI, starch, and fat. Feng et al. [24] found similar aggregation constructed by a starch and pea protein network through SEM. Although starch was the major ingredient in their study, they demonstrated that an increasing amount of pea protein began to establish a continuous pea protein matrix. The 20CHK sample exhibits a layered structure, but no fibrous structure was observed. The visible aligned fibrous structure in the 50CHK was observed inside the plant protein-based matrices (Fig. 2). A similar fibrous structure is also found in cooked chicken mince. The structure of cooked chicken mince was very similar to chicken meat under SEM in previous studies [13, 19]. It indicates that cooked chicken mince exhibits fibers.

These findings suggest that the fibrous structure is created by chicken directly. PPI or other ingredients would not provide fiber through the preparation and printing process in this study. The large proportion of chicken paste in the 50CHK samples assists in forming obvious fibers. However, such structure also negatively influenced the extrusion smoothness, resulting in poor printing performance with few fibers presented in the macrostructure [15]. To print a fibrous non-meat product, other potential plant-based fiber-forming agents need to be added. More research on fiber-forming mechanisms is required.

### Protein Solubility

**Fig. 3:**
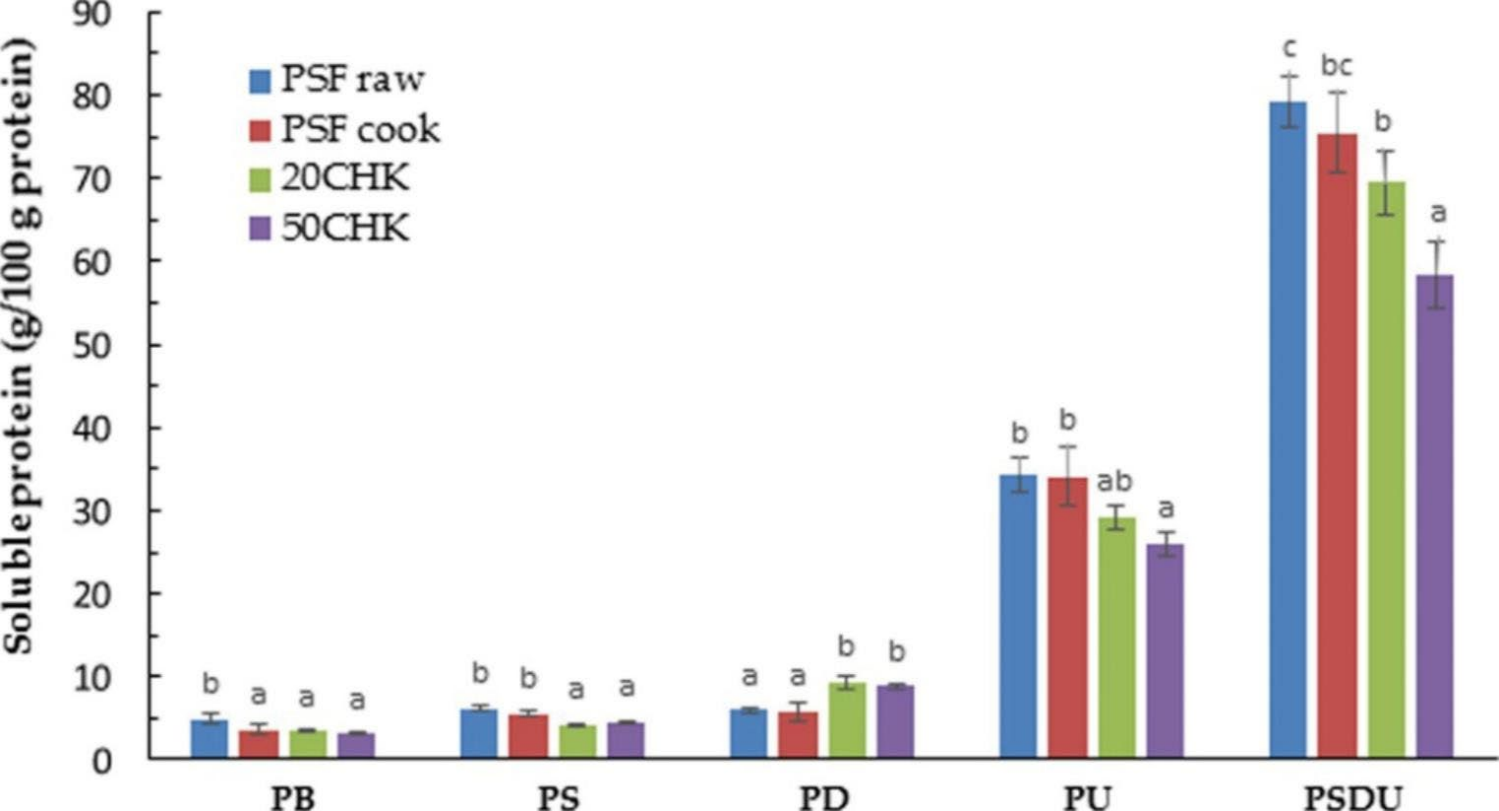
Soluble protein percentages in PSF paste, Printed PSF, 20CHK and 50CHK in five extraction solutions Each bar shows means ± SD (*n* = 3). Data with different letters for each extraction treatment are significantly different (*p <* 0.05). PSF raw represents PPI paste containing starch and fat without cooking; Printed PSF represents PPI containing starch and fat after cooking; 20CHK represents 20% chicken added into PSF paste; 50CHK represents 50% chicken added into PSF paste. PSF cook, 20CHK, and 50CHK samples were printed and cooked in boiling water for 10 min. PB represents phosphate buffer; PS represents PB + SDS; PD represents PB + DTT; PU represents PB + urea; PSDU represents PB + SDS + DTT + urea.

Protein solubility was found to be the lowest in PB compared with all extraction solutions (Fig. 3). It indicates that protein interactions exist and supports the structure of samples. Even though the PSF paste showed a significantly higher solubility than the other three samples in PB (*p <* 0.05), it was still lower than 5%. Such low solubility further proves that the PPI used in this study had already been denatured during the manufacturing process since only native plant protein can be dissolved in a phosphate buffer [9]. It was obvious that soluble protein in PU was higher than in PS and PD, demonstrating that proteins are more soluble in urea than in SDS and DTT. It is believed that urea destroys hydrogen bonds [25]. Thus, hydrogen bonding is considered the major interaction between proteins in all samples. Previous research also showed that hydrogen bonds were the major force in pea legume protein gel [26].

Protein-protein interactions have been recognized as a mechanism of fiber formation. The formation of fibrous structures is associated with the formation of hydrophobic interactions, disulfide bonds among proteins, or the combination of both bonds [27]. Extruded meat analogs tended to show a high protein solubility in DTT solvents, as it breaks disulfide bonding [9, 13]. In this study, the disulfide bond is also considered as the main protein-protein interaction responsible for fibrous structures. This is because chicken-added samples, which have a fibrous structure, showed lower protein solubility in overall extraction solutions except in DDT-containing buffer solution (PD). However, PSF paste and printed PSF did not show significantly different (*p <* 0.05) protein solubility in PD, interpreted as showing that printing and cooking did not help form fibers. It has already been discussed that fibers in printed samples were mainly provided by chicken paste. Similar to the results observed by Chiang et al. [12], the protein solubility of all samples in PSDU is greater than the sum of solubility in PS, PD, and PU. It indicates that the structure of PSF paste and printed meat analogs are not only supported by hydrophobic interactions, disulfide, and hydrogen bonds but also their combinations.

## Conclusions

The meat-like fibrous structure was not clearly observed in the PPI-only sample. It is recognized that fibers in printed hybrid meat analogs are contributed by native chicken muscle fibers. Nevertheless, adding a higher amount of chicken (50CHK) decreased the extrudability, which also negatively influenced the printing performance, resulting in fewer fibers shown in the macrostructure. Through the protein solubility assay, it was known that hydrogen bonding is the major protein interaction that helps support the structure of printed meat analogs. The disulfide bonding was observed to be the main interaction related to fibrous structure. Printed meat analogs showed a higher softness compared with conventionally cooked chicken meat.

For this purpose, it is highly recommended to conduct sensory studies on 3D-printed meat analogues. People with mastication issues or who are interested in soft foods could be selected and trained as sensory panellists.

## Electronic Supplementary Material

Below is the link to the electronic supplementary material.

Supplementary Material 1

## Data Availability

The data may be made available upon request.
