# Supplementary material for: Physico-chemical and Textural Properties of 3D Printed Plant-based and Hybrid Soft Meat Analogs
Source: Plant Foods Hum Nutr. 2023 May 18;78(2):375–82. doi: 10.1007/s11130-023-01068-4 (PMC10363036; doi:10.1007/s11130-023-01068-4)
Supplement: Supplementary file 1 — Supplementary Material 1 [file 11130_2023_1068_MOESM1_ESM.docx]

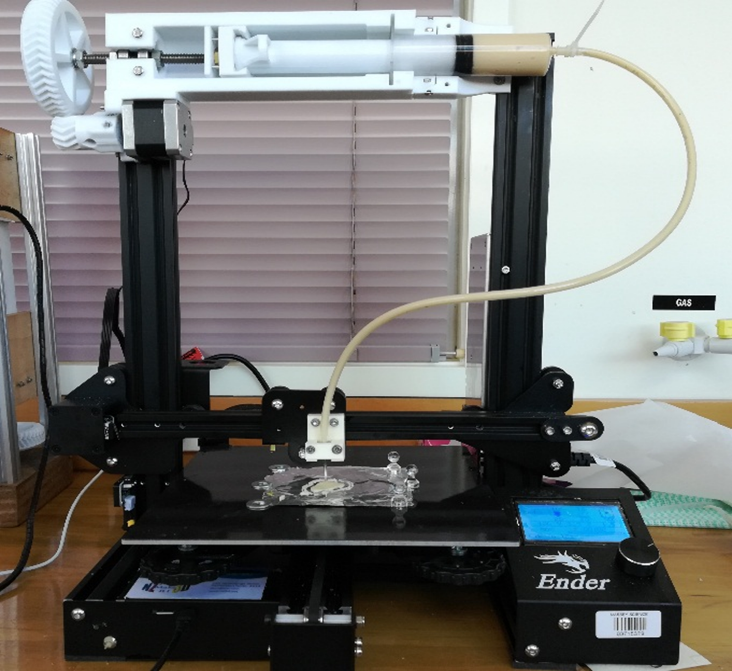


*Supplementary Figure 1* A photo of the 3D printer set-up used in the study.

It comprised an extruder unit, motor, nozzle holder, platform, and conveyor belts, controlling the movement to x, y, and z-axis directions, operation menu, a USB connection to a computer, and framework of Ender-3 3D printer.

# Abbreviations

3D Three dimensional

AOAC Association of Official Agricultural Chemists

BSA Bovine serum albumin

PPI Pea protein isolate

SEM Scanning electron microscopy

TPA Texture profile analysis
